# AR Pathway Is Involved in the Regulation of CX43 in Prostate Cancer

**DOI:** 10.1155/2015/514234

**Published:** 2015-09-28

**Authors:** Ruibao Chen, Yang Luan, Zhuo Liu, Wen Song, Licheng Wu, Mingchao Li, Jun Yang, Xiaming Liu, Tao Wang, Jihong Liu, Zhangqun Ye

**Affiliations:** ^1^Department of Urology, Tongji Hospital, Tongji Medical College, Huazhong University of Science and Technology, Wuhan, Hubei 430030, China; ^2^Institute of Urology, Tongji Hospital, Tongji Medical College, Huazhong University of Science and Technology, Wuhan, Hubei 430030, China

## Abstract

CX43 plays a critical role in tumor progression. Previous studies imply that AR pathway may be involved in regulation of CX43. This study was focused on determining the relationship between AR pathway and CX43. The result showed that the expression of CX43 in malignant cells was higher than that in normal cells, and in nonmalignant and malignant cells, not only is the expression level of CX43 different, but the localization of CX43 can also be changed. After androgen stimulation and inhibition of AR pathway, expression of CX43 can also be changed. Thus, AR pathway plays an important role in regulation of CX43 expression in prostate cancer cells. AR may be the upstream signal of CX43.

## 1. Introduction

Prostate cancer (PCa) is the most commonly diagnosed male cancer in the Western world [1]. Localized early-stage PCa can be well treated by radical prostatectomy. By contrast, advanced PCa is mainly treated with androgen deprivation therapy (ADT), but most cases will eventually fail ADT, recur, and result in a lethal disease termed castration resistant prostate cancer (CRPC) [2]. The etiology for PCa development and molecular mechanisms underlying castration resistant progression are incompletely understood. Currently, a lot of evidence demonstrated that the development of prostate cancer is closely related with the AR pathway, but the exact mechanism is unclear.

Connexins are widely expressed transmembrane proteins that are known to form gap junction channels. They play critical roles in cell growth, tissue regeneration, and carcinogenesis. As a member of gap junction channels, connexin 43 (CX43) was also found to be disregulated in multiple types of cancers and participate in tumor progression, including gastric, cervical, colorectal, and prostate cancers. In breast cancer, the downregulation of CX43 significantly promoted tumor progression, while overexpression of CX43 could suppress the cancer phenotype of human mammary carcinoma cells and restore differentiation potential, suggesting that CX43 can function as a tumor suppressor [3].

Previous studies also confirmed that the expression and positioning of CX43 are closely related to pathological progression of the PCa [4]. CX43 expression had a significant change in the late/high aggressive prostate cancer cells, and the expression level decreasing was negatively correlated with pathological grading [4]. Cell localization of CX43 showed apparent translocation in the highly invasive PCa cell lines. In normal prostate epithelial cells, CX43 mainly located in the cell membrane with a functional state. But in high aggressive PCa cells, CX43 mainly located in the cytoplasm with a nonfunctional state. Studies had shown that CX43 protein localized in cytoplasm in nonfunctional state when the PC-3 cells were overexpressing CX43. Animal xenograft model and in vitro cell culture studies confirmed that overexpression of CX43 would inhibit proliferation, differentiation, and invasion of PC-3 cells. As a tumor suppressor factor, CX43 and GJIC function missing mediated by CX43 played an important role in PCa development [5], but the upstream molecular biological basis of CX43 in prostate cancer cells is still poorly understood.

In order to demonstrate that the expression and localization of CX43 were associated with androgen receptor (AR) pathway, we conducted a series of experiments. Our studies revealed that CX43 expression level is higher in malignant cells than in nonmalignant cells. Activation of AR pathway represses CX43 expression. Inhibition of AR pathway increased the expression of CX43. Taken together, our findings reveal a tumor suppressive mechanism for AR/CX43 pathway and established CX43 as molecular determinant in androgen deprivation therapy for PCa.

## 2. Materials and Methods

### 2.1. Cell Lines, Antibodies, and Channel Blockers

RWPE-1, LNCaP, PC-3, and 22RV1 cell lines were purchased from China Center for Type Culture Collection. CX43 and AR antibody were purchased from SANTA CRUZ. CX43siRNA and negative control were purchased from GenePharma. Lipofectamine RNAiMAX Reagent and Opti-MEM Reduced Serum Medium were purchased from Invitrogen. Cell Counting Kit-8 (CCK-8) was purchased from DOJINDO. ASC-J9 was purchased from AdooQ BioScience. Charcoal stripped fetal bovine serum and fetal bovine serum were purchased from Invitrogen. Keratinocyte-SFM were purchased from Gibco.

### 2.2. Western Blot Analysis

Western blot assay was done as previously described [6]. Cells were treated as indicated in figures. LNCaP and 22RV1 cells were cultured in DMED/F12 and 10% charcoal stripped fetal bovine serum overnight before experiments as previously described [6]. Cells were washed and pelleted in cold phosphate-buffered saline and lysed on ice in RIPA buffer. Equal amount of proteins from each lysate was loaded onto SDS-PAGE gels and transferred onto PVDF membrane. The membranes were incubated with the primary antibodies and secondary antibodies. After several washes, bands were revealed by enhanced chemiluminescence detection system.

### 2.3. Immunofluorescence and Confocal Laser Scanning

Immunofluorescence assay was done as previously described [7]. Cells were treated as indicated in figures. LNCaP cells were cultured in DMED/F12 and 10% charcoal stripped fetal bovine serum overnight before experiments as previously described [6]. Cells were washed with phosphate-buffered saline and fixed with 4% paraformaldehyde in PBS for 30 minutes. Cells were then immunostained with the primary antibodies and secondary antibodies indicated in figures. Finally, the slides were scanned under an Olympus FV1000 confocal laser microscope.

### 2.4. CCK-8 Assay

CCK-8 assay was done according to the manufacturer's instructions. LNCaP cells were treated as indicated in figures. After the indicated time, 10 *µ*L CCK-8 solution was added to each well and incubated for 2 h at 28°C; after that the absorbance was measured at 450 nm using a microplate reader. Experiments were repeated in triplicate.

### 2.5. Statistical Analysis

All values are presented as means ± standard deviation (SD). The significance of the differences between treatment and control was analyzed using SPSS software.

## 3. Results

### 3.1. The Expression and Location of CX43 Are Different among Normal and Tumor Cells

**Figure 1:**
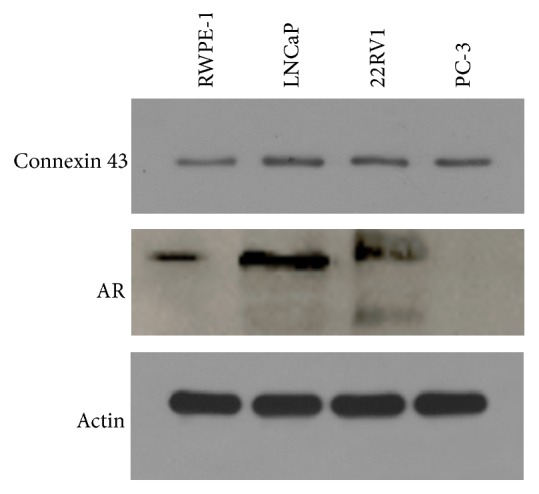
Expression of CX43 and AR in RWPE-1, LNCaP, 22RV1, and PC-3 cells was detected by western blot.

Previous studies reported that CX43 was downregulated in several cancers [8]. In order to detect the expression of CX43 in prostate cancer cells, we surveyed the commonly used prostate cancer cell lines for CX43. As shown in Figure 1, the expression of CX43 in malignant cells was higher than that in normal cells. We then investigate the localization of CX43 in malignant cells and normal cells by detecting CX43 in cytoplasm and cell membrane, respectively. As shown in Figure 3(a), in nonmalignant RWPE-1 cells, the expression level of CX43 in cytoplasm and cell membrane is rarely low, but in malignant LNCaP cells, the expression level of CX43 in cytoplasm and cell membrane is much higher. CX43 was mainly located in cell membrane in nonmalignant RWPE-1 cells and in cell cytoplasm in malignant LNCaP cells. These results indicate that, in nonmalignant and malignant cells, not only is the expression level of CX43 different, but the localization of CX43 can also be changed.

### 3.2. Activation of AR Pathway Inhibits the Expression of CX43 in Prostate Cancer Cells

**Figure 2:**
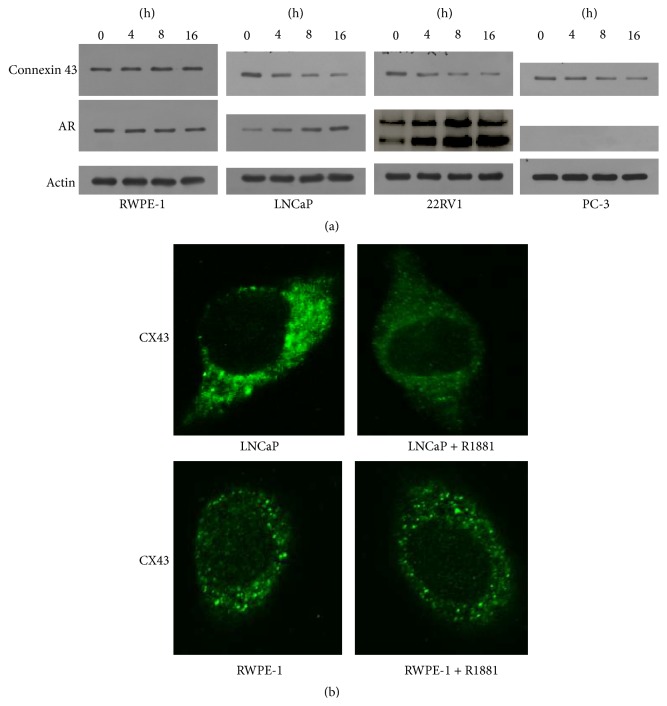
Expression of CX43 after androgen intervention. (a) Expression of CX43 was detected by western blot in RWPE-1, LNCaP, 22RV1, and PC-3 cells after R1881 2 nM treatment in time course. (b) Expression of CX43 in RWPE-1 and LNCaP cells was detected by immunofluorescence and confocal laser scanning after R1881 2 nM treatment for 24 h.

Previous report showed that androgen downregulated CX43 in human granulosa cells [9]. To determine the relationship between CX43 and AR pathway in prostate cancers, we detected the expression of CX43 in nonmalignant and malignant prostate cells after androgen stimulated in time course. As shown in Figure 2(a), in LNCaP and 22RV1 cells, the expression of CX43 was downregulated as early as androgen stimulation for 8 hours, and the expression of CX43 constantly decreased with the extension of time. But in the nonmalignant RWPE-1 cells and PC-3 (AR negative) cells the expression of CX43 almost had no change.

Next, we detected the expression of CX43 by confocal assay after staining of CX43. These results were consistent with previous studies. The expression of CX43 in LNCaP cells was downregulated after androgen stimulation, but there was no similar change in RWPE-1 cells (Figure 2(b)).

### 3.3. Inhibition of AR Pathway Increases the Expression of CX43, While CX43 Knockdown Could Partially Abolish the Effect of Blocking AR Pathway

**Figure 3:**
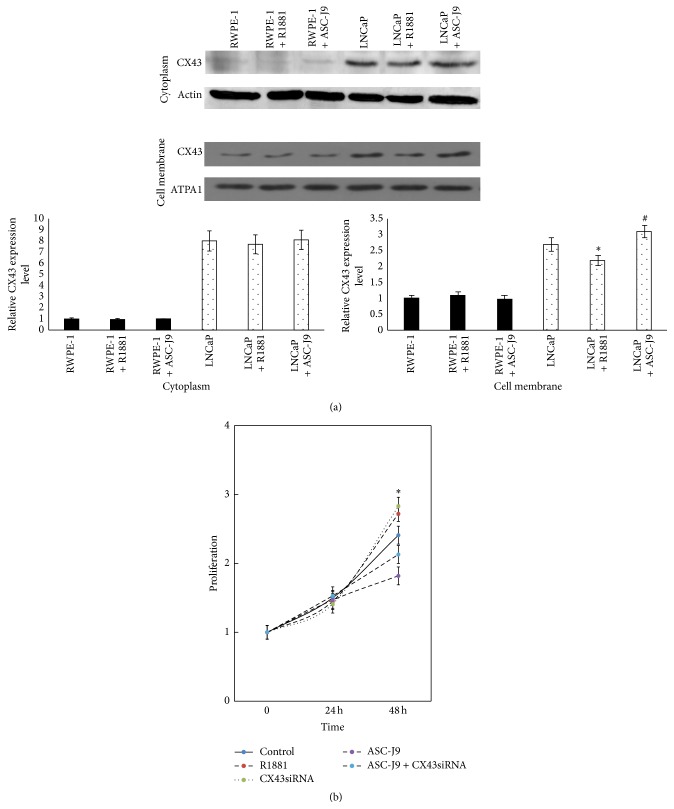
Analysis of the relationship between AR pathway and CX43. (a) In RWPE-1 and LNCaP cells, expression of CX43 in cytoplasm and cell membrane was detected by western blot, respectively, after R1881 2 nM or ASC-J9 10 *µ*M treatment for 24 h. Graphical presentation showing pixel intensities of indicated group. Data were obtained in three independent experiments and are represented as mean ± SD. ^*∗*, #^ *p* value is <0.05 compared to control. (b) Proliferation of LNCaP cells was detected by CCK-8 assay after CX43siRNA 100 nM or/and ASC-J9 10 *µ*M treatment in time course as indicated. Data were obtained in three independent experiments and are represented as mean ± SD. ^*∗*^ *p* value is <0.05 compared to control.

Previous studies showed that the expression level and localization of CX43 in prostate cancer cells were different from nonmalignant prostate cells. We enhanced AR pathway with androgen and inhibited AR pathway with ASC-J9 which could degrade both full-length AR (fAR) and AR3 [10]. Then we detected the expression of CX43 in cell membrane and cytoplasm after regulation of AR pathway. The result showed that the expression of CX43 was significantly increased after ASC-J9 treatment and significantly decreased after androgen treatment in cell membrane in LNCaP cells, but there were no obvious differences in cytoplasm after ASC-J9 and androgen treatment (Figure 3(a)).

Finally, we detected the proliferation of LNCaP cells after regulation of AR pathway and CX43 expression by CCK-8 assay; the result showed that inhibition of CX43 expression promotes the proliferation of LNCaP cells and blocking AR pathway could suppress cell proliferation, if we knock down the expression of CX43, which could partially reverse the effect of blocking AR pathway (Figure 3(b)).

## 4. Discussion

Many biological phenomena are vital for cancer cells. By focusing on the cellular aspects, we advocate a holistic view of cancer—how functional networks, cellular interactions, and microenvironmental factors affect carcinogenesis. This contrasts with a reductionist view of cancer, which emphasizes the molecular aspects, such as genetic aberrations and signaling pathways [1]. Connexins are a group of homologous proteins that form the intermembrane channels of gap junctions. An abnormal expression level and distribution of connexins are closely related to tumor formation [11]. However, the functional network of connexins remains not clear.

CX43 is the most widely expressed gap junction protein and closely related to biological processes of cancer. Expression of CX43 was significantly downregulated in colon cancer and overexpression of CX43 can directly increase gap junction activity [8, 12]. In this study, we found that the expression level of CX43 in prostate cancer cells was higher than that in nonmalignant prostate cells. The cohort study revealed that tumors with positive surgical margins showed significantly lower CX43 expression compared with tumors without this feature in prostate cancer [4]. These studies indicate that the role of CX43 in prostate cancer may be different from its role in other tumors due to the unique biological characteristics of prostate cancer.

Much has been learned about the pivotal role of the AR in more than 70 years since androgens were first identified as the major drivers of prostate cancer. More work still needs to be performed to understand the exact mechanisms of AR pathway. It is possible that multiple different mechanisms may be at work [13]. Based on the special role of AR in prostate cancer and the difference of CX43 expression in prostate cancer and other tumors, we examined the effect of AR pathway on CX43. The results showed that androgen can inhibit CX43 expression in prostate cancer cells, but this phenomenon is not obvious in nonmalignant prostate cells. And the location of CX43 in prostate cancer cell is different from that in nonmalignant prostate cells. Activating AR pathway mainly inhibits CX43 expression in prostate cancer cell membrane, and CX43 expression of cell membrane in nonmalignant prostate cells is at low level, which just provide the answer that AR pathway poorly affects CX43 expression in nonmalignant prostate cells. Later, the cell proliferation assay revealed that downregulated CX43 expression can partially abolish the effect of inhibition of AR pathway. These results indicated that CX43 is closely related to AR pathway and AR may be the upstream signal of CX43.

Recently, studies reported that AR may function as both suppressor and proliferator to suppress or promote prostate cancer metastasis [14]. The expression and location of CX43 are different in prostate cancer and nonmalignant prostate cells. And in different kinds of cancers, the correlation between CX43 and cancer is different. These bodies of evidence suggest that AR may participate in the bidirectional regulation of CX43. However, the exact regulation mechanism between AR and CX43 remains to be further studied.

## 5. Conclusion

This study demonstrates that the expression level and location of CX43 in malignant and nonmalignant prostate cells are different. CX43 is closely related to AR pathway and AR may be the upstream signal of CX43. AR may participate in the bidirectional regulation of CX43. However, the exact regulation mechanism between AR and CX43 remains to be further studied.
